# Establishment of Three Different Glycation-Damage Cell Models and Analysis of Their Action Mechanism

**DOI:** 10.3390/metabo16050346

**Published:** 2026-05-21

**Authors:** Xinya Qian, Chencan Cao, Lei Liu

**Affiliations:** College of Light Industry Science and Engineering, Beijing Technology and Business University, Higher Education Garden, Liangxiang, Fangshan District, Beijing 102488, China; qianxinya720@163.com (X.Q.); caochencan99@163.com (C.C.)

**Keywords:** glycation, cell model, untargeted metabolomics, anti-glycation effect

## Abstract

**Background/Objective:** With rising per capita sugar consumption, skin glycation-related issues including dullness, homeostasis disruption and accelerated wrinkling have gained widespread attention. However, globally standardized and rigorous evaluation criteria for anti-glycation efficacy remain lacking. This study aimed to establish stage-specific glycation injury cell models and elucidate the stage-dependent molecular mechanisms of glycation-induced fibroblast damage, providing a standardized reference for anti-glycation efficacy assessment. **Methods:** Three glycation injury models were constructed in human foreskin fibroblasts (HFF-1): early-stage (glucose-induced), intermediate-stage (glyoxal-induced), and late-stage (advanced glycation end products (AGEs)-induced). Core biomarkers including Nε-(carboxymethyl)lysine (CML), collagen type I (Col I) and elastin (ELN) were used to optimize modeling conditions via Cell Counting Kit-8 (CCK-8) and enzyme-linked immunosorbent assay (ELISA). Untargeted metabolomics based on ultra-high-performance liquid chromatography (UHPLC)-Q Exactive Orbitrap was applied to identify differential metabolites and perturbed pathways, following Metabolomics Standards Initiative (MSI) Level 2 identification criteria. **Results:** Optimal conditions were determined as 50 mmol/L glucose for 48 h, 0.5 mmol/L glyoxal for 48 h, and 200 μg/mL AGEs for 24 h. A total of 319, 34 and 148 differential metabolites were identified in the three groups, respectively. Six key pathways were significantly perturbed. Early and intermediate models shared similar mechanisms (purine metabolism disturbance), while the late model showed distinct alterations in pyrimidine, nicotinate, arachidonic acid and steroid hormone metabolism. **Conclusions:** Three stable stage-specific glycation models were successfully established in HFF-1 cells. Significant differences in metabolic profiles and mechanisms exist across the three stages, providing a rational basis for model selection and theoretical support for anti-glycation efficacy evaluation.

## 1. Introduction

With improvements in living standards and increasing per capita sugar intake, public interest in the occurrence of glycation and strategies to combat glycation has increased significantly. Glycation, also referred to as non-enzymatic glycation (NEG), is a chemical process wherein the carbonyl groups of reducing sugars react with the N-terminal amino groups of macromolecules (e.g., proteins, nucleic acids, and lipids). These initial adducts subsequently undergo oxidation, rearrangement, and cross-linking reactions, ultimately leading to the progressive accumulation of irreversible advanced glycation end products (AGEs) [1].

The glycation reaction is generally divided into three sequential stages: early, intermediate, and late [2,3]. In the early stage, reducing sugars react with amino groups of proteins to form reversible Schiff bases (unstable imine intermediates), which then rearrange into Amadori products (ketoamine derivatives). In the intermediate stage, Amadori products undergo further reactions to generate dicarbonyl compounds. In the late stage, these intermediates may undergo Strecker degradation via condensation with free amino acids, forming imines that cleave to produce Strecker aldehydes and ultimately leading to the formation of AGEs. Glycation accelerates skin aging through multiple mechanisms, such as disrupting extracellular matrix (ECM) components, regulating matrix metalloproteinase expression, promoting cell apoptosis, and upregulating the receptor for AGEs (RAGE) to exacerbate inflammatory responses [4].

Although numerous methods and standards have been established for evaluating cosmetic efficacy, a set of scientifically rigorous and effective approaches for assessing anti-glycation efficacy has not yet been standardized domestically or internationally. Dermal fibroblasts are the major cellular component of the dermis; in addition to supporting epidermal migration, proliferation, and differentiation, they synthesize and secrete key ECM constituents (e.g., collagen and elastin) and multiple reparative factors. These functions enable fibroblasts to maintain and restore cutaneous homeostasis, making them central to anti-glycation responses. Therefore, based on the mechanistic process of glycation, we used human foreskin fibroblasts (HFF-1) to establish three stage-specific glycation injury models corresponding to the early, intermediate, and late phases of glycation. This study aims to clarify the mechanisms underlying glycation-induced skin damage, compare stage-dependent differences, and provide recommendations for the future development of standardized cell-based models for evaluating anti-glycation efficacy.

## 2. Materials and Methods

### 2.1. Reagent and Materials

Human foreskin fibroblasts (HFF-1, Cat. No. BNCC100406) were obtained from BNCC (Beijing, China). High-glucose DMEM (4.5 g/L, Cat. No. C11995500BT), fetal bovine serum (Cat. No. 10099141), and penicillin–streptomycin (Cat. No. 15140-122) were purchased from Gibco (Waltham, MA, USA). Advanced glycation end products (AGEs, glycolaldehyde-modified AGE-BSA, Cat. No. BN40025) were obtained from Biorigin (Naperville, IL, USA). Glucose (Cat. No. G8270) was purchased from Lablead (Beijing, China). Aminoguanidine hydrochloride (Cat. No. A151036) was purchased from Aladdin (Shanghai, China). Cell counting kit-8 (CCK-8) assay kit (Cat. No. C0038) and bicinchoninic acid (BCA) protein assay kit (Cat. No. BN27109) were supplied by Biorigin (Naperville, IL, USA). Human Nε-(carboxymethyl)lysine (CML) enzyme-linked immunosorbent assay (ELISA) kits (Cat. No. CSB-E12798h), Human collagen type I (ColI) ELISA kit (Cat. No. CSB-E08082h) and Human elastin (ELN) ELISA kit (Cat. No. CSB-E09338h) were purchased from CUSABIO (Wuhan, China). Liquid chromatography-tandem mass spectrometry (LC-MS) grade methanol (Cat. No. 106035) and acetonitrile (Cat. No. 100030) were obtained from Merck (Darmstadt, Germany). Acetic acid (Cat. No. R000030) was purchased from Rhawn (Shanghai, China). Formic acid (Cat. No. F112038), ammonium formate (Cat. No. A100188), and ammonia solution (Cat. No. A112084) were purchased from Aladdin (Shanghai, China). Analytical reference standards with purity ≥98.0% were obtained from Sigma-Aldrich (St. Louis, MO, USA), TCI (Tokyo, Japan), TRC (Toronto, ON, Canada) and ISOREAG (Shanghai, China).

### 2.2. Cell Culture

HFF-1 were cultured in high-glucose Dulbecco’s Modified Eagle’s Medium (DMEM) supplemented with 10% fetal bovine serum (FBS) and 1% penicillin–streptomycin. Cells were maintained at 37 °C in a humidified incubator with 5% CO_2_ and routinely passaged when reaching approximately 90% confluence for subsequent experiments.

### 2.3. Cell Viability Assay

Cells were seeded in 96-well plates at a density of 6 × 10^4^ cells/mL for the early- and middle-stage glycation models, or 8 × 10^4^ cells/mL for the late-stage glycation model, and incubated for 24 h. Following group allocation, 100 μL of DMEM containing test substances at various concentrations was added to each well. The cells were then incubated for an additional 48 h in the early- and middle-stage models, or 24 h in the late-stage model. Cell viability was determined using a CCK-8 kit by measuring the optical density (OD) at 450 nm.

### 2.4. ELISA

Cells for the early- and middle-stage glycation models were seeded in 6-well plates at a density of 6 × 10^4^ cells/mL, while those for the late-stage glycation model were seeded at 8 × 10^4^ cells/mL, followed by incubation for 24 h. According to group assignments, 2 mL of DMEM supplemented with test substances at different concentrations was added to each well. The early- and middle-stage model cells were incubated for a further 48 h, whereas the late-stage model cells were incubated for an additional 24 h. The levels of NE-CML in cell lysates were quantified by ELISA kits at 450 nm. The total protein concentration of cell lysates was determined via BCA assay at 562 nm for data calibration. The contents of Col I and ELN in cell culture supernatants were measured simultaneously using ELISA kits at 450 nm without total protein normalization.

### 2.5. Untargeted Metabolomics

#### 2.5.1. Sample Preparation

Cells were seeded in 10 cm dishes at 8 × 10^4^ cells/mL (10 mL per dish) and cultured for 24 h. To establish early, middle and late glycation models, cells were treated with glucose, glyoxal (GO) and AGEs, respectively, and further incubated for 48 h (glucose and GO groups) or 24 h (AGE group). After discarding the medium, cells were rinsed twice with ice-cold PBS, scraped into ice-cold PBS, and collected by centrifugation at 1000× *g* for 5 min at 4 °C. Cells were washed twice more with ice-cold PBS under the same centrifugation conditions. Subsequently, 500 μL of 80% aqueous methanol containing 2 μg/mL 2-chloro-L-phenylalanine (internal standard) was added, and samples were vortexed for 30 s. Three liquid nitrogen–37 °C freeze–thaw cycles were performed, with 30 s vortexing after each thaw. After centrifugation at 12,000× *g* for 10 min at 4 °C, 300 μL supernatant was collected, incubated at −20 °C for 30 min for protein precipitation, and re-centrifuged at 12,000× *g* for 3 min at 4 °C. Finally, 200 μL of the supernatant was transferred into an autosampler vial for LC-MS analysis.

#### 2.5.2. LC-MS/MS Analysis

Untargeted metabolomic profiling of cell samples was performed on a Thermo Fisher Ultimate 3000 UHPLC system coupled to a Q Exactive Orbitrap mass spectrometer (Thermo Fisher Scientific, Waltham, MA, USA). Chromatographic separation was carried out on a Waters ACQUITY Premier HSS T3 column (1.8 μm, 2.1 mm × 100 mm; Waters Corporation, Milford, MA, USA). Mobile phase A was water containing 0.1% formic acid, and mobile phase B was acetonitrile with 0.1% formic acid. The injection volume was 4 μL, column temperature was maintained at 40 °C, and the flow rate was 0.4 mL/min. The gradient elution program is listed in Table 1.

#### 2.5.3. Data Processing

Raw LC-MS data were processed using the XCMS package in R for peak detection, peak alignment, and retention time correction. Metabolite annotation and identification were conducted by matching accurate mass, MS/MS fragmentation patterns, and retention time against an in-house database, public databases (HMDB, METLIN, LipidMaps), and the MetDNA in silico prediction platform. Non-biological xenobiotic candidates irrelevant to human cellular metabolism were manually excluded to eliminate systematic identification bias. Metabolites with a composite identification score >0.5 and coefficient of variation (CV) <0.3 in quality control (QC) samples were retained. All annotated metabolites were further assigned to confidence levels following the Metabolomics Standards Initiative (MSI) criteria, and all identified differential metabolites were uniformly designated as MSI Level 2. Positive and negative ionization data were integrated to generate a comprehensive metabolite profile, and metabolomic intensities were normalized to BCA-determined total protein content to eliminate sample loading differences.

QC sample correlation analysis evaluated instrumental stability and data reproducibility. Unsupervised three-dimensional principal component analysis (3D-PCA) and supervised orthogonal partial least squares discriminant analysis (OPLS-DA) assessed metabolic separation among groups, with Welch’s *t*-test for pairwise comparisons. Differential metabolites were screened by variable importance in the projection (VIP) >1.0, fold change (FC) >1.5 or <0.667, and *p* < 0.05, with Benjamini–Hochberg FDR correction. OPLS-DA model validity was confirmed by 200 random permutations. Venn diagrams characterized shared/unique differential metabolites, and hierarchical clustering heatmaps visualized their expression patterns and similarity (shorter branches = higher similarity). All computational analyses were performed in R with dedicated metabolomics packages.

### 2.6. Statistical Analysis

All experimental data are presented as mean ± standard deviation (SD). Statistical analysis was performed using GraphPad Prism 9.0. One-way ANOVA followed by Tukey’s multiple comparison test was applied for conventional cellular experiments, with the statistical significance level set at *p* < 0.05. The significance levels were marked as *p* < 0.05, *p* < 0.01, *p* < 0.001, and *p* < 0.0001.

## 3. Results

### 3.1. Establishment of the Early Glycation Damage Model

**Figure 1:**
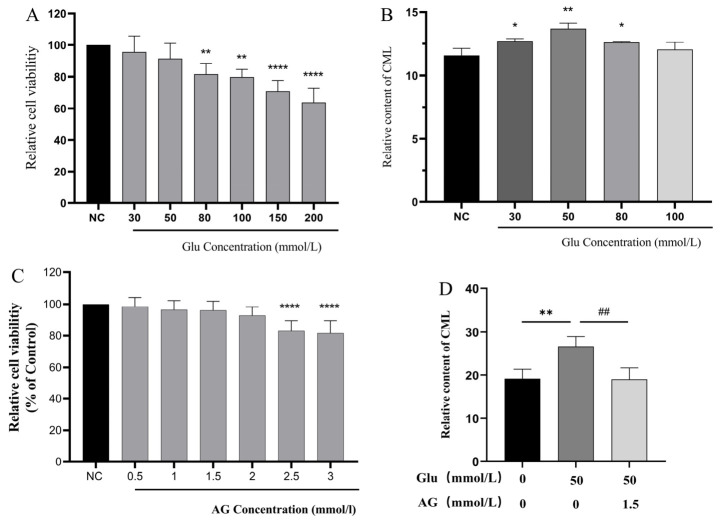
Optimization of glucose-induced early glycation modeling conditions and positive control selection. (**A**) Effects of different concentrations of glucose on HFF-1 cell viability (cell counting kit-8 (CCK-8) assay); (**B**) Effects of different concentrations of glucose on Nε-(carboxymethyl)lysine (CML) content (ELISA assay); (**C**) Effects of different concentrations of aminoguanidine (AG) on HFF-1 cell viability (CCK-8 assay); (**D**) Effects of 1.5 mmol/L AG on CML content in 50 mmol/L glucose-induced HFF-1 cells (enzyme-linked immunosorbent assay (ELISA) assay). Data are presented as mean ± standard deviation (SD). Data presented are from one experiment with 6 technical replicates for CCK-8 assays and 3 technical replicates for ELISA assays. Data are presented as mean ± SD. * *p* < 0.05, ** *p* < 0.01, **** *p* < 0.0001compared with the normal control (NC) group; ## *p* < 0.01 compared with the model group.

High glucose promotes the activation of the receptor for advanced glycation end products (RAGE), triggers the accumulation of AGEs, and further induces cellular senescence and apoptosis [5]. High-concentration glucose is widely employed as a classic inducer in glycation modeling, which mainly acts on the early stage of the glycation reaction [6,7]. CML is one of the most prevalent advanced glycation end products (AGEs) in organisms, which is often used as a representative AGE to explore the physiological toxicity, formation pathway and inhibition mechanism of endogenous AGEs [8]. Aminoguanidine is regarded as a typical anti-glycation agent. It mainly functions at the intermediate stage of glycation reaction and traps α-dicarbonyl intermediates through nucleophilic reaction with carbonyl-scavenging activity, and has been widely used in related studies [9]. The effects of different glucose concentrations on HFF-1 cell viability and CML levels were evaluated. As illustrated in Figure 1A, cell viability declined in a concentration-dependent manner with elevated glucose levels. A statistically significant decrease in cell viability relative to the NC group first emerged at 80 mmol/L (*p* < 0.01). Nevertheless, cell viability at 30, 50, 80 and 100 mmol/L was still no less than 79.82% ± 4.16%, with the lowest value recorded at 100 mmol/L, all staying above the 80% viability threshold. Concentrations of 150 mmol/L and above markedly lowered cell viability below the threshold (*p* < 0.0001 vs. NC). Moreover, CML content peaked at 50 mmol/L glucose (13.67 ± 0.3146; Figure 1B). Accordingly, 50 mmol/L glucose intervention for 48 h was determined as the optimal condition to establish the HFF-1 cell glycation model.

Based on a literature review, guanidine was selected as the positive control. Therefore, the effect of different concentrations of guanidine on HFF-1 cell viability and CML content was measured. The results indicated that cell viability remained above 92.82% ± 5.03% at concentrations ranging from 0.5 to 2 mmol/L (Figure 1C), without significant effects on cell viability, showing no cytotoxicity of guanidine. At a concentration of 1.5 mmol/L, the CML content/total protein concentration was significantly reduced (Figure 1D), indicating that 1.5 mmol/L guanidine has a repair effect on early-stage glycation damage in HFF-1 cells and can be used as a positive control.

### 3.2. Establishment of the Middle-Stage Glycation Damage Model

**Figure 2:**
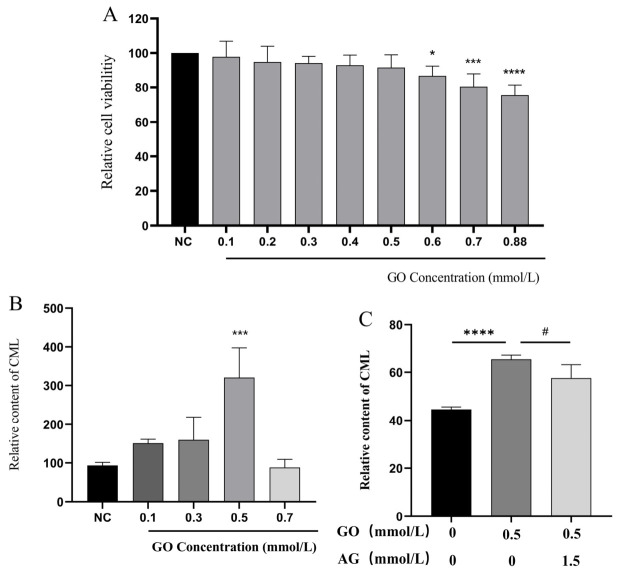
Optimization of glyoxal-induced intermediate glycation modeling conditions and positive control selection. (**A**) Effects of different concentrations of glyoxal on HFF-1 cell viability (CCK-8 assay). (**B**) Effects of different concentrations of glyoxal on Nε-(carboxymethyl)lysine (CML) content (ELISA assay). (**C**) Effects of 1.5 mmol/L AG on CML content in glyoxal-induced HFF-1 cells (ELISA assay). Data are presented as mean ± SD. Data presented are from one experiment with 6 technical replicates for CCK-8 assays and 3 technical replicates for ELISA assays. * *p* < 0.05, *** *p* < 0.001, **** *p* < 0.0001 vs. the NC group; # *p* < 0.05 vs. the model group.

GO, a typical reactive carbonyl species (RCS), is a cellular metabolic byproduct that damages proteins, nucleic acids and lipids and contributes to aging and chronic disorders [10]. Serving as a key glycating agent and AGE precursor, GO reacts with protein residues to accelerate AGE generation. AG acts as a classic synthetic AGE inhibitor by scavenging α-dicarbonyl intermediates via nucleophilic reactions during the middle phase of glycation, thereby suppressing AGE deposition, and is extensively adopted in glycation research [9,11]. The effects of different GO concentrations on HFF-1 cell viability and CML content were evaluated. The results indicated that at concentrations of 0.1, 0.3, 0.5, and 0.7 mmol/L, the cell viability remained ≥80.34% ± 6.76% (at 0.7 mmol/L) (Figure 2A). The CML content reached its peak at a concentration of 0.5 mmol/L (Figure 2B). Therefore, the final modeling condition was set as 0.5 mmol/L GO to stimulate HFF-1 cells for 48 h.

The effect of the previously identified 1.5 mmol/L aminoguanidine concentration on CML content was measured in this model. The results showed that the CML content/total protein concentration was significantly reduced (48.04 ng/mg ± 4.6707, *p* < 0.05) (Figure 2C), indicating that 1.5 mmol/L of AG has a repair effect on the HFF-1 cells with intermediate glycation damage, and thus can be used as a positive control.

### 3.3. Establishment of the Late-Stage Glycation Damage Model

**Figure 3:**
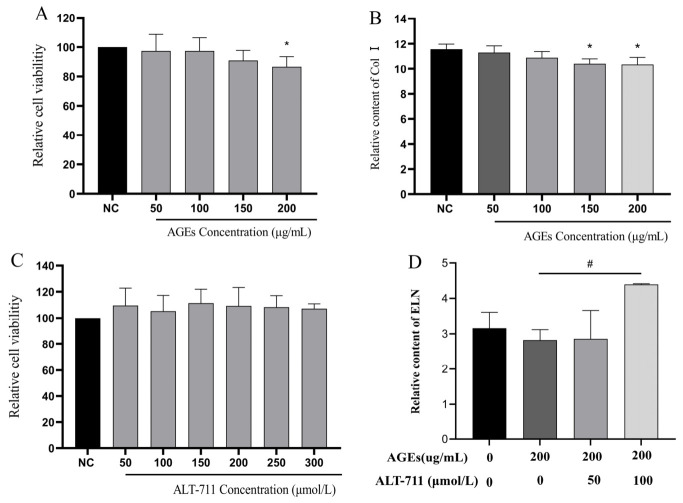
Optimization of AGE-induced late glycation modeling conditions and positive control selection. (**A**) Effects of different concentrations of AGEs on HFF-1 cell viability (CCK-8 assay). (**B**) Effects of different concentrations of AGEs on type Col I content (ELISA assay). (**C**) Effects of different concentrations of alagebrium chloride (ALT-711) on HFF-1 cell viability (CCK-8 assay). (**D**) Effects of different concentrations of ALT-711 on ELN content in 200 μg/mL AGEs-induced HFF-1 cells (ELISA assay). Data are presented as mean ± SD. Data presented are from one experiment with 6 technical replicates for CCK-8 assays and 3 technical replicates for ELISA assays. * *p* < 0.05, vs. the NC group; # *p* < 0.05 vs. the model group.

AGE-modified bovine serum albumin (AGE-BSA) is a glycation product formed by the reaction of bovine serum albumin (BSA) with reducing sugars or their derivatives under sterile conditions, which induces cellular glycation injury at the late stage of the glycation cascade. In the in vitro preparation of AGEs, BSA is the predominant protein substrate, and AGEs derived from BSA and glucose (or glucose derivatives) are the most commonly used glycation inducers in current studies [12]. Alagebrium chloride (ALT-711) serves as a typical AGE breaker that mainly functions at the late stage of glycation, disrupting AGE-protein crosslinks and regulating the AGE-RAGE signaling pathway [13]. Col I and ELN are core structural proteins in the skin ECM, which are highly susceptible to glycation-induced dysfunction and serve as key markers for evaluating glycation injury and repair efficacy [14]. The effects of different advanced glycation end product (AGE) concentrations on HFF-1 cell viability and Type I collagen levels were determined. The results showed that at concentrations ranging from 50 to 200 μg/mL, the cell viability remained ≥86.49% ± 6.53% (at 200 μg/mL) (Figure 3A). The Type I collagen content in the cell supernatant/total protein concentration decreased in a dose-dependent manner (Figure 3B). Therefore, the final modeling condition was set as 200 μg/mL AGEs to stimulate HFF-1 cells for 24 h.

Based on a literature review, ALT-711 was selected as the positive control. Therefore, the effect of different concentrations of ALT-711 on HFF-1 cell viability and ELNcontent was measured. The results showed that within the concentration range of 50 to 300 μmol/L, there was no significant change in cell viability (Figure 3C). At a concentration of 100 μmol/L, the ELN content in the cell supernatant/total protein concentration was significantly increased (Figure 3D), indicating that 100 μmol/L ALT-711 has a repair effect on HFF-1 cells with advanced glycation damage and can be used as a positive control.

### 3.4. Screening of Differential Metabolites

**Figure 4:**
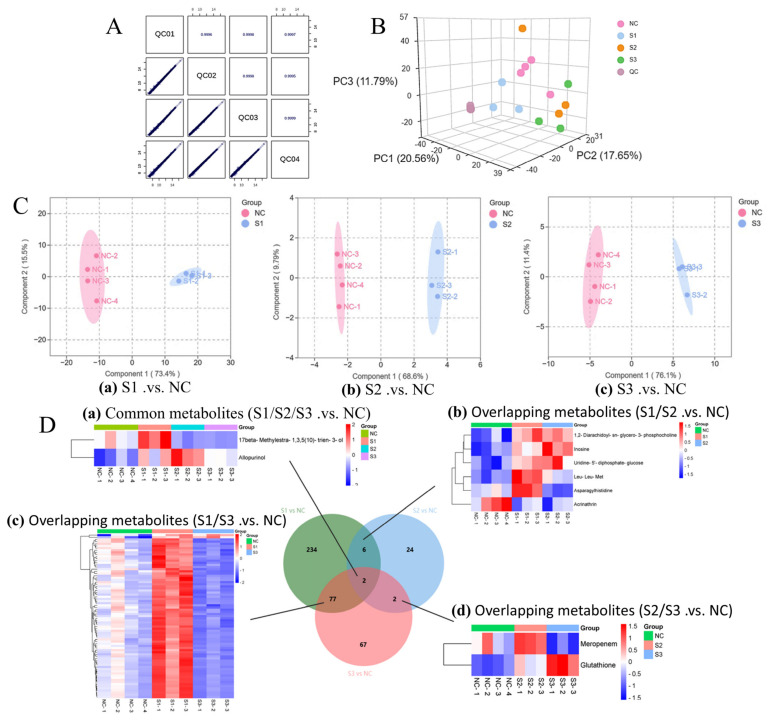
Screening of differential metabolites. (**A**) Pearson correlation heatmap of quality control (QC) samples. Four QC samples were prepared by pooling equal volumes of all samples and injected at the beginning, middle, and end of the liquid chromatography-tandem mass spectrometry (LC-MS) run to evaluate instrumental stability and data reproducibility. (**B**) Three-dimensional principal component analysis (3D-PCA) score plot of all samples, showing the overall metabolic separation among groups. (**C**) Orthogonal partial least squares discriminant analysis (OPLS-DA) score plots for pairwise comparisons: (**a**) S1 vs. NC, (**b**) S2 vs. NC, and (**c**) S3 vs. NC. The coefficient of determination (R^2^Y) and predictive correlation coefficient (Q^2^) represent the explained variance and predictive capability of the OPLS-DA model, respectively. (**D**) Venn diagram and hierarchical clustering heatmaps of differential metabolites. (**a**–**d**) Venn diagrams and corresponding hierarchical clustering heatmaps of overlapping differential metabolites identified from pairwise comparisons: (**a**) common metabolites shared by S1.vs.NC, S2.vs.NC, and S3.vs.NC; (**b**) metabolites shared by S1.vs.NC and S2.vs.NC; (**c**) metabolites shared by S1.vs.NC and S3.vs.NC; (**d**) metabolites shared by S2.vs.NC and S3.vs.NC. Differential metabolites were screened with VIP >1.0, fold change >1.5 or <0.667, *p* < 0.05, and Benjamini–Hochberg FDR correction. Rows indicate metabolites and columns indicate samples; red and blue represent higher and lower metabolite abundance, respectively. Metabolites are clustered along the row axis, with shorter dendrogram branches indicating higher similarity in expression patterns. Group definitions: NC, normal control group (*n* = 4); S1, glucose-treated group (*n* = 3); S2, glyoxal-treated group (*n* = 3); S3, AGEs-treated group (*n* = 3). QC samples were inserted every 5 injections throughout the run, and QC-RLSC correction was used to minimize signal drift.

Multivariate statistical analysis was performed on the preprocessed metabolomic data for sample classification. First, Pearson correlation analysis of QC samples (Figure 4A) and principal component analysis (PCA) of all samples (including QC samples, Figure 4B) were conducted to evaluate sample reproducibility and overall metabolic similarity across groups. To further characterize metabolic differences among the normal control (NC), glucose (Glu), glyoxal (GO), and AGEs-treated groups, OPLS-DA was applied for pairwise comparison (Figure 4C(a–c)). Clear separation was observed between the NC group and each glycation injury group, verifying the reliability of the established cell models and indicating distinct metabolic profiles under different glycation stimuli.

Differential metabolites were screened using three statistical criteria: VIP, FC, and *p*-value. The screening thresholds were set as VIP > 1.0, FC > 1.5 or FC < 0.667, and *p* < 0.05. The detailed screening results are presented in Table 2.

The differential metabolites from S1.vs.NC, S2.vs.NC, and S3.vs.NC were integrated using a Venn diagram for further analysis. A summary table (Table 3) and a visualization of the results (Figure 4D) are provided. All metabolites were normalized, and the single overlapping differential metabolite identified from the screening was visualized using a clustered heatmap (Figure 4D).

### 3.5. Analysis of Overlapping Differential Metabolites Among Groups

#### 3.5.1. Overlapping Differential Metabolites in Glu, GO, and AGEs Injury Groups

Two overlapping differential metabolites were shared across the glucose, glyoxal, and AGEs intervention groups, namely 17β-methylestra-1,3,5(10)-trien-3-ol and allopurinol. These metabolites were categorized into alcohols and nucleotide-related metabolites, respectively (Table 4).

Notably, the metabolite annotated as allopurinol does not correspond to the exogenous antigout drug. In untargeted metabolomics profiling, endogenous purine intermediates frequently display highly consistent accurate mass and MS/MS fragmentation patterns with allopurinol, leading to automatic database matching under spectral similarity scoring. This annotated feature is deemed an endogenous purine structural analog in HFF-1 cells. Its altered abundance indicates perturbed purine metabolism induced by glycation stress, rather than the actual existence of exogenous allopurinol in cell samples.

#### 3.5.2. Overlapping Differential Metabolites in Glu and GO Injury Groups

Five overlapping differential metabolites were shared by the Glu and GO groups. These metabolites mainly belonged to small peptides, phosphatidylcholines, and nucleotide metabolites (Table 5).

#### 3.5.3. Overlapping Differential Metabolites in Glu and AGEs Injury Groups

A total of 75 overlapping differential metabolites was screened between the Glu and AGE groups. They were primarily distributed in multiple metabolic categories, including peptides, amino acid metabolites, free fatty acids, alcohols and derivatives, ketone derivatives, and heterocyclic compounds. A small proportion were assigned to vitamins and cofactors, alkaloids, oxidized lipids and esters. Partial information of these metabolites is summarized in Table 6.

#### 3.5.4. Overlapping Differential Metabolites in GO and AGEs Injury Groups

One common differential metabolite was found in the GO and AGE groups: glutathione and meropenem. They belong to amino acid metabolites and organic acid derivatives, respectively (Table 7).

It is noteworthy that a small fraction of annotated metabolites was matched to exogenous drugs or antibiotics via public database alignment. This represents an inherent technical limitation of untargeted LC-MS/MS metabolomics. Numerous endogenous small molecules, lipids, and structural isomers in HFF-1 cells possess nearly identical molecular mass and fragmentation profiles to certain exogenous compounds. Databases assign the most probable compound name primarily based on spectral similarity, which does not verify the physical presence of such exogenous substances in the tested samples. These annotations are generally endogenous structural analogs, and their abundance alterations reflect metabolic dysfunction, oxidative stress, and inflammatory responses triggered by glycation injury.

### 3.6. Pathway Analysis of Differential Metabolites

**Figure 5:**
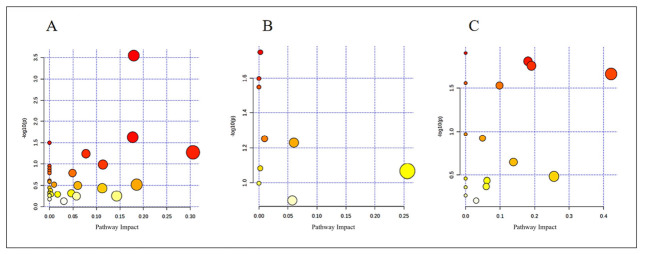
Metabolic Pathway Analysis of Differential Metabolites. (**A**) Glu vs. NC; (**B**) GO vs. NC; (**C**) AGEs vs. NC. Interpretation guide: X-axis: Pathway impact value, calculated by topological analysis, representing the relative importance of the pathway in the entire metabolic network. Y-axis: −log10 (*p* value), representing the statistical significance of pathway enrichment. The horizontal dashed line indicates the significance threshold of *p* = 0.05. Bubble size: Proportional to the number of differential metabolites identified in the corresponding pathway. Bubble color: Gradient from yellow to red indicates increasing enrichment significance.

To identify and visualize the impacted metabolic pathways, pathway enrichment analysis was performed separately for the differential metabolites of each comparison group using MetaboAnalyst 6.0 (Figure 5). Detailed results are provided in Table 8, Table 9 and Table 10. The S1.vs.NC comparison involved 31 metabolic pathways (Figure 5A); the S2.vs.NC comparison involved 9 pathways (Figure 5B); and the S3.vs.NC comparison involved 16 pathways (Figure 5C).

Metabolic pathways with *p* < 0.05 and an impact value >0 from the above analyses were selected as the major pathways significantly impacted in Glu/GO/AGEs-induced HFF-1 cell injury. For the Glu injury group (S1), the major pathways were Purine Metabolism and Sphingolipid Metabolism. For the GO injury group (S2), the major pathway was Purine Metabolism. For the AGE injury group (S3), the major pathways were Pyrimidine Metabolism, Nicotinate and Nicotinamide Metabolism, Arachidonic Acid Metabolism, and Steroid Hormone Biosynthesis.

## 4. Discussion

In this study, three cell models corresponding to early, intermediate and late glycation damage were established using HFF-1 fibroblasts.

For the early-stage model, 50 mM glucose was administered for 48 h to induce early glycation, with CML selected as the core evaluation biomarker. This strategy effectively recapitulated the early glycation process, and 1.5 mM aminoguanidine was applied as the positive control to alleviate cellular damage. Elevated glucose levels accelerate the glycation process and promote the progression of the downstream AGEs cascade [15,16]. High-glucose stimulation also facilitates intracellular AGE accumulation and exacerbates glycation injury in skin cells [17]. The glucose concentration and treatment duration adopted herein are consistent with classic protocols for constructing early glycation models in fibroblasts [4], confirming the rationality and reproducibility of our model system.

For the intermediate-stage model, 0.5 mM glyoxal (GO) was used to induce intermediate glycation injury in HFF-1 cells for 48 h. CML was also adopted as the biological marker, and 1.5 mM aminoguanidine served as the positive control. This intervention successfully mimicked the phenotypic characteristics of intermediate glycation. The glyoxal concentration used in this study falls within the optimal range for triggering intermediate glycation in dermal fibroblasts [10], and the treatment duration and biomarker selection are consistent with mainstream research protocols [4], further validating the reliability of this model.

For the late-stage model, HFF-1 cells were stimulated with 200 μg/mL AGEs for 24 h, with type I collagen chosen as the evaluation biomarker. A dose of 100 μmol/L ALT-711 was used as the positive control, which effectively rescued late glycation-associated cellular damage. Both the AGEs treatment concentration and incubation duration complied with well-recognized modeling conditions for late glycation in skin cells [10,17]. In addition, the 100 μmol/L ALT-711 selected in our study is consistent with the effective in vitro dose reported to reverse AGE cross-linking [13].

It should be noted that the three glycation models adopted different induction durations (24 h and 48 h), which were determined by the inherent kinetic properties of each inducer rather than arbitrary experimental design. Glucose and GO trigger a gradual intracellular non-enzymatic glycation cascade, including Schiff base formation, Amadori rearrangement and dicarbonyl generation; a 48 h intervention is required to establish stable early- and intermediate-stage glycation injury. In contrast, exogenous AGEs are mature terminal glycation products that directly activate the RAGE pathway without dependence on intracellular stepwise biosynthesis. A 24 h incubation is sufficient to induce reliable late-stage damage, while prolonged 48 h treatment causes excessive cellular injury and a marked decline in cell viability [4,10].

Notably, the early and intermediate models received identical 48 h incubation but exhibited distinct metabolic profiles and pathway perturbations. PCA and OPLS-DA clustering clearly separated samples according to glycation stage rather than incubation time, verifying that the observed molecular differences originated from stage-dependent glycation effects, rather than variations in treatment duration.

Furthermore, the 50 mM glucose used in this study is a classic supraphysiological concentration widely adopted for in vitro early glycation modeling, which does not correspond to the physiological hyperglycemic level in vivo (typically 10–20 mM). Although high glucose slightly increases medium osmolarity, the alterations in cell viability, CML content and metabolic profiles are mainly attributed to glucose-triggered glycation and oxidative stress, rather than hyperosmotic stress. The prominent protective effect of aminoguanidine further confirms that the biological changes stem from glycation injury rather than osmotic interference [5,7].

Here we clarify the rationale for classifying the three models into early, intermediate and late glycation stages, and further validate that they represent a unified sequential glycation cascade rather than three independent biological processes. First, the staging strictly follows the canonical non-enzymatic glycation pathway: glucose initiates early glycation through Schiff base and Amadori product formation; glyoxal functions as a key dicarbonyl intermediate driving the intermediate glycation phase; exogenous AGEs mimic irreversible protein crosslinking in the late stage [2,3,4]. Second, core biomarkers including CML, type I collagen and elastin exhibited progressive, stage-dependent changes across the three models, which align with the natural temporal progression of glycation damage. Third, PCA and OPLS-DA showed a continuous gradient separation from the normal control group to early, intermediate and late models. The early and intermediate groups shared overlapping differential metabolites and consistent perturbation of purine metabolism, while the late model presented unique pathway characteristics. Collectively, these results confirm that the three models recapitulate successive phases of an integrated glycation cascade, rather than three unrelated biological processes.

A total of two common metabolites were shared among early-, middle-, and late-stage glycation injury groups (Table 4), namely 17β-methyl-1,3,5(10)-estratrien-3-ol and allopurinol. The abundance of 17β-methyl-1,3,5(10)-estratrien-3-ol displayed distinct alteration patterns under different stimulations: it was upregulated in the glucose group but downregulated in the glyoxal and AGE groups, implying its predominant involvement in the early glycation response [14,15]. The metabolite annotated as allopurinol showed the lowest abundance in the normal control group and was differentially upregulated after glucose, glyoxal and AGEs intervention, with the most pronounced increase observed in the glyoxal-treated group. As an endogenous purine structural analog annotated via spectral matching, its elevation reflects the disruption of purine metabolism under glycation stress. Relevant evidence indicates that purine metabolite perturbation can modulate NF-κB signaling and immune homeostasis in skin keratinocytes, implying that different stages of glycation injury may interfere with cutaneous immune regulation by reshaping intracellular purine metabolic profiles [18].

Five overlapping metabolites were identified between early- and middle-stage glycation groups (Table 5). L-leucine-L-leucine-L-methionine, a bioactive small peptide, acts as a vital signaling molecule and participates in protein–protein interaction regulation across biological systems [19]. Inosine, a purine nucleoside containing hypoxanthine, can be generated via adenine hydrolytic deamination in mammalian cells [20]. UDP-glucose functions as a key glucose donor for glycosylation reactions and biosynthesis of bioactive compounds [21]. 1,2-Dipalmitoyl-sn-glycerol-3-phosphocholine is a major membrane phospholipid, which maintains cell membrane integrity and modulates multiple intracellular signaling cascades [21]. The levels of these four metabolites were significantly upregulated after glucose and glyoxal intervention. The elevated glucose/glyoxal may increase the supply of protein precursors, glucose donors, nucleotides and lipids. The interaction of glucose/glyoxal with biological macromolecules (proteins, nucleic acids and lipids) further accelerates the progression of glycation reactions [1,15,21].

A total of 75 metabolites overlapped between early- and late-stage glycation groups (Table 6). Among them, methyl stearate and 2-oleoylglycerol were markedly upregulated under glucose and AGEs stimulation. As a typical glycerolipid, 2-oleoylglycerol participates in lipid storage and transport [22]. It is hypothesized that glucose and AGE treatment accelerates intracellular glycation, elevates energy consumption, and promotes lipid transport to sustain the progression of glycation processes. The remaining 73 overlapping metabolites exhibited opposite expression trends: they were upregulated in the glucose-induced early glycation group but downregulated in the AGE-induced late glycation group. These metabolites mainly modulate the core glycation process of HFF-1 cells, with limited involvement in downstream events such as AGE–collagen crosslinking and receptor–ligand interaction-mediated signaling reprogramming. Based on previous reports, these differential metabolites are speculated to primarily regulate the AGEs/RAGE, Mitogen-Activated Protein Kinase (MAPK), NF-κB and collagen metabolism pathways in fibroblasts [17,23,24,25]. In the early glycation triggered by high glucose, upregulated metabolites mainly initiate intracellular glycation and mild oxidative stress without excessive activation of downstream inflammatory and fibrotic pathways. In contrast, the downregulated metabolites in the AGE group are closely linked to persistent AGEs/RAGE axis activation, which further induces MAPK cascade phosphorylation and NF-κB nuclear translocation, ultimately aggravating collagen crosslinking, inflammatory responses and functional impairment of skin fibroblasts [8,17,24,25].

One metabolite was shared between middle- and late-stage glycation groups (Table 7). Glutathione is a multifunctional molecule with antioxidant properties, which regulates DNA synthesis and repair, protects protein thiol groups, stabilizes cell membranes and mediates xenobiotic detoxification [26]. Omics analysis showed that glutathione abundance was the lowest in the control group and significantly upregulated in glyoxal and AGE groups. It is speculated that glyoxal and AGE-induced glycation injury triggers massive ROS accumulation in HFF-1 cells, which further stimulates intracellular glutathione synthesis as a compensatory antioxidant response.

In the early glycation model, purine metabolism and sphingolipid metabolism were markedly disturbed. In purine metabolism, glucose intervention upregulated multiple differential metabolites including L-glutamine, 5′-phosphoribosylglycinamide, IMP, inosine and adenosine, triggering purine metabolic disorder. Such metabolic perturbation aggravates oxidative stress and promotes glycation-associated skin aging [20,27,28]. In sphingolipid metabolism, glucose increased the levels of sphingosine, dihydroceramide and phytosphingosine, facilitating ceramide synthesis and precursor accumulation in HFF-1 cells [29,30,31]. This perturbs sphingolipid homeostasis, enhances cell apoptosis and oxidative stress, and ultimately accelerates glycation-induced premature skin aging [32,33,34].

The middle-stage glycation model also exhibited obvious dysregulation of purine metabolism. GO stimulation elevated inosine and guanosine abundance, leading to purine metabolic disturbance, oxidative stress exacerbation and subsequent glycation injury [20,27,28].

By contrast, the late-stage AGE model showed abnormal alterations in pyrimidine metabolism, nicotinate and nicotinamide metabolism, arachidonic acid metabolism, and steroid hormone biosynthesis.

In pyrimidine metabolism, AGEs increased 2′-deoxycytidine-5′-monophosphate while decreasing 5-uracil nucleotides, which induced pyrimidine synthesis disorder, aggravated apoptosis and impaired mitochondrial function [35,36].

In nicotinate and nicotinamide metabolism, AGEs suppressed the levels of nicotinic acid mononucleotide and imidazole aspartate, disturbed pathway homeostasis and reduced NAD production, thereby promoting cellular senescence and death [37,38]. In arachidonic acid metabolism, AGEs downregulated arachidonic acid, prostaglandin G2 and leukotriene B4, which aggravated oxidative stress, disrupted immune balance and triggered inflammatory responses [8,39,40,41].

In steroid hormone biosynthesis, multiple metabolites including androstenedione, testosterone and estrone were decreased upon AGE treatment. This induces hormonal imbalance, inhibits collagen synthesis, amplifies oxidative stress and inflammation, and accelerates fibroblast aging [8,17].

Pathway analysis revealed consistent purine metabolic dysregulation in both early and middle models, implying shared underlying mechanisms of glucose- and glyoxal-induced glycation damage. However, the late AGE model displayed distinct metabolic pathway signatures, indicating that the glycation injury mechanism triggered by AGEs differs substantially from that induced by glucose and glyoxal.

Early and middle models partially recapitulate the cellular functional alterations during the initial glycation process, whereas the late model better mimics the damage caused by macromolecular crosslinking products derived from non-enzymatic glycation.

In summary, this study established three classic skin glycation models induced by glucose, GO and AGEs, which are widely adopted in anti-glycation research. Despite partial similarities, the three models present distinct metabolic characteristics corresponding to different glycation stages and action mechanisms. Our findings provide a rational reference for the selection of appropriate in vitro glycation models according to research purposes.

## 5. Conclusions

This study successfully established early-, intermediate- and late-stage glycation injury models in HFF-1 fibroblasts by using glucose, glyoxal and AGEs as inducers, respectively, and optimized the modeling conditions and positive control concentrations based on cell viability and characteristic biomarker levels. Untargeted metabolomics analysis showed that early and middle glycation models shared obvious disturbance in purine metabolism, while the late-stage model displayed distinct metabolic alterations in pyrimidine metabolism, nicotinate and nicotinamide metabolism, arachidonic acid metabolism and steroid hormone biosynthesis. Obvious differences in metabolic profiles and molecular mechanisms existed among the three glycation stages. This work provides stable and standardized cell models for anti-glycation research, and clarifies the stage-specific mechanisms of glycation-induced fibroblast damage, offering a theoretical basis for the evaluation of anti-glycation and skin anti-aging efficacy.

## Figures and Tables

**Table 1:** Gradient elution program for the HSS T3 column.

Time (min)	A (%)	B (%)
0.0	95	5
2.0	80	20
5.0	40	60
6.0	1	99
7.5	1	99
7.6	95	5
10.0	95	5

Note: Mobile phase A: water containing 0.1% formic acid; Mobile phase B: acetonitrile containing 0.1% formic acid.

**Table 2:** Statistical summary of differential metabolites and the number of upregulated and downregulated metabolites in each group.

Group	Metabolites	Upregelated	Downregulated
S1.vs.NC	319	310	9
S2.vs.NC	34	21	13
S3.vs.NC	148	26	122

**Table 3:** Statistical summary of overlapping differential metabolites and the number of upregulated and downregulated metabolites in each Comparison group.

Comparison	Metabolites	Same	Opposite
S1.vs.NC and S2.vs.NC	6	4	2
S1.vs.NC and S3.vs.NC	77	2	75
S2.vs.NC and S3.vs.NC	2	1	1
S1.vs.NC, S2.vs.NC and S3.vs.NC	2	1	1

**Table 4:** Differential Screening Results of Overlapping Metabolites in S1 vs. NC, S2 vs. NC, and S3.vs.NC.

Names	Class	S1.vs.NC	S2.vs.NC	S3.vs.NC	MSI Confidence Level
17beta-Methylestra-1,3,5(10)-trien-3-ol	Alcohol	up	down	down	Level 2
Allopurinol	Nucleotide and Its metabolites	up	up	up	Level 2

**Table 5:** Differential Screening Results of Overlapping Metabolites in S1 vs. NC, S2 vs. NC.

Names	Class	S1.vs.NC	S2.vs.NC	MSI Confidence Level
Uridine-5′-diphosphate-glucose	Nucleotide and Its metabolites	up	up	Level 2
1,2-Diarachidoyl-sn-glycero-3-phosphocholine	PC	up	up	Level 2
Leu-Leu-Met	Small Peptide	up	up	Level 2
Inosine	Nucleotide and Its metabolites	up	up	Level 2
Asparagylhistidine	Small Peptide	up	down	Level 2

**Table 6:** Differential Screening Results of Overlapping Metabolites in S1 vs. NC, S3 vs. NC.

Names	Class	S1.vs.NC	S3.vs.NC	MSI Confidence Level
Methyl stearate	FA	up	up	Level 2
2-Linoleoylglycerol	MG	up	up	Level 2
Val-Leu-Leu-Val-Val	Small Peptide	up	down	Level 2
Ala-Arg-Pro-Lys-Leu	Small Peptide	up	down	Level 2
Arachidonic acid	FFA	up	down	Level 2
Octyl-Beta-D-Glucopyranoside	Sugars	up	down	Level 2
Glimepiride	polypeptide	up	down	Level 2
Terpendole E	Heterocyclic compounds	up	down	Level 2
Asn-Gln-His	Small Peptide	up	down	Level 2
Lys-Gln-Ala-Gly-Asp-Val	Small Peptide	up	down	Level 2
Iminoaspartic acid	Amino acids	up	down	Level 2
LTB4	Oxidized lipids	up	down	Level 2
D-phenylalanine	Amino acids	up	down	Level 2
2,5-Dimethyl-3-(methyldithio)furan	Heterocyclic compounds	up	down	Level 2
Pyridoxine phosphate	Vitamin	up	down	Level 2
Bovinic acid	Oxidized lipids	up	down	Level 2
FFA(22:6)	FFA	up	down	Level 2
FFA(22:5)	FFA	up	down	Level 2
FFA(18:1)	FFA	up	down	Level 2
Eicosapentaenoic acid	FFA	up	down	Level 2
Cytarabine	Nucleotide and Its metabolites	up	down	Level 2
Tyr-Ser-Phe-Val-Phe	Small Peptide	up	down	Level 2
Asp-His-Phe-Asp	Small Peptide	up	down	Level 2
Aspartylasparagine	Small Peptide	up	down	Level 2
Val-Gln-Ala-Arg	Small Peptide	up	down	Level 2

**Table 7:** Differential Screening Results of Overlapping Metabolites in S2 vs. NC, S3 vs. NC.

Names	Class	S2.vs.NC	S3.vs.NC	Annotation Confidence
Glutathione	Amino acid derivatives	up	up	Level 2

**Table 8:** Metabolic pathway analysis of biomarkers for differential metabolites in Glu vs. NC.

	Total	Expected	Hits	Raw *p*	Homl Adjust	FDR	Impact
Purine metabolism	70	1.3778	7	0.00028384	0.022707	0.022707	0.17956
Sphingolipid metabolism	32	0.62984	3	0.023281	1	0.84755	0.17719
Biosynthesis of unsaturated fatty acids	36	0.70857	3	0.031783	1	0.84755	0
Arachidonic acid metabolism	44	0.86603	3	0.053041	1	0.91479	0.30572
Citrate cycle (TCA cycle)	20	0.39365	2	0.057174	1	0.91479	0.07771
Alanine, aspartate and glutamate metabolism	28	0.55111	2	0.10329	1	1	0.11378
Nitrogen metabolism	6	0.1181	1	0.1126	1	1	0
Glyoxylate and dicarboxylate metabolism	32	0.62984	2	0.1292	1	1	0
Valine, leucine and isoleucine biosynthesis	8	0.15746	1	0.14734	1	1	0
Vitamin B6 metabolism	9	0.17714	1	0.1642	1	1	0
Ascorbate and aldarate metabolism	9	0.17714	1	0.1642	1	1	0
Arginine biosynthesis	14	0.27556	1	0.24382	1	1	0
Butanoate metabolism	15	0.29524	1	0.25883	1	1	0
Nicotinate and nicotinamide metabolism	15	0.29524	1	0.25883	1	1	0
Terpenoid backbone biosynthesis	18	0.35429	1	0.30217	1	1	0
Starch and sucrose metabolism	18	0.35429	1	0.30217	1	1	0.00974
Pentose and glucuronate interconversions	19	0.37397	1	0.31607	1	1	0.06024
Propanoate metabolism	22	0.43302	1	0.35616	1	1	0
Pentose phosphate pathway	23	0.4527	1	0.36901	1	1	0.1124
Galactose metabolism	27	0.53143	1	0.41798	1	1	0.00228
Porphyrin metabolism	31	0.61016	1	0.46326	1	1	0
Glycine, serine and threonine metabolism	33	0.64952	1	0.48461	1	1	0.04653
Cysteine and methionine metabolism	33	0.64952	1	0.48461	1	1	0
Glycerophospholipid metabolism	36	0.70857	1	0.51509	1	1	0.01736
Steroid hormone biosynthesis	87	1.7124	2	0.51893	1	1	0.00403
Pyrimidine metabolism	39	0.76762	1	0.54382	1	1	0
Valine, leucine and isoleucine degradation	40	0.7873	1	0.55303	1	1	0
Tryptophan metabolism	41	0.80698	1	0.56206	1	1	0.14305
Amino sugar and nucleotide sugar metabolism	42	0.82667	1	0.57091	1	1	0.05755
Drug metabolism—cytochrome P450	55	1.0825	1	0.67134	1	1	0
Metabolism of xenobiotics by cytochrome P450	68	1.3384	1	0.74884	1	1	0.03061

**Table 9:** Metabolic pathway analysis of potential biomarker metabolites screened from differential metabolites in the GO vs. NC comparison.

	Total	Expected	Hits	Raw *p*	Homl Adjust	FDR	Impact
Purine metabolism	70	0.22222	2	0.017852	1	0.75419	0.00245
Valine, leucine and isoleucine biosynthesis	8	0.025397	1	0.025172	1	0.75419	0
Ascorbate and aldarate metabolism	9	0.028571	1	0.028282	1	0.75419	0
Starch and sucrose metabolism	18	0.057143	1	0.055921	1	0.94324	0.00974
Pentose and glucuronate interconversions	19	0.060317	1	0.058953	1	0.94324	0.06024
Galactose metabolism	27	0.085714	1	0.082927	1	0.98159	0.00228
Glutathione metabolism	28	0.088889	1	0.085889	1	0.98159	0.25596
Glycine, serine and threonine metabolism	33	0.10476	1	0.10059	1	1	0
Amino sugar and nucleotide sugar metabolism	42	0.13333	1	0.12656	1	1	0.05755

**Table 10:** Metabolic pathway analysis of potential biomarker metabolites screened from differential metabolites in the AGEs vs. NC comparison.

	Total	Expected	Hits	Raw *p*	Homl Adjust	FDR	Impact
Biosynthesis of unsaturated fatty acids	36	0.50286	3	0.012535	1	0.39447	0
Pyrimidine metabolism	39	0.54476	3	0.015614	1	0.39447	0.18072
Nicotinate and nicotinamide metabolism	15	0.20952	2	0.017525	1	0.39447	0.19079
Arachidonic acid metabolism	44	0.6146	3	0.021625	1	0.39447	0.42155
Neomycin, kanamycin and gentamicin biosynthesis	2	0.027937	1	0.02775	1	0.39447	0
Steroid hormone biosynthesis	87	1.2152	4	0.029585	1	0.39447	0.09816
Valine, leucine and isoleucine biosynthesis	8	0.11175	1	0.10666	1	1	0
Vitamin B6 metabolism	9	0.12571	1	0.1192	1	1	0.04902
Starch and sucrose metabolism	18	0.25143	1	0.22476	1	1	0.13851
Glutathione metabolism	28	0.39111	1	0.32787	1	1	0.25596
Inositol phosphate metabolism	30	0.41905	1	0.34686	1	1	0
Sphingolipid metabolism	32	0.44698	1	0.36533	1	1	0.06191
Drug metabolism—other enzymes	39	0.54476	1	0.42615	1	1	0.05978
Valine, leucine and isoleucine degradation	40	0.55873	1	0.43436	1	1	0
Drug metabolism—cytochrome P450	55	0.76825	1	0.54495	1	1	0
Metabolism of xenobiotics by cytochrome P450	68	0.94984	1	0.6238	1	1	0.03061

## Data Availability

The original contributions presented in this study are included in the article. Further inquiries can be directed to the corresponding author.

## Abbreviations

The following abbreviations are used in this manuscript:

AGEs	advanced glycation end products
AG	aminoguanidine
ALT-711	Alagebrium chloride
BCA	bicinchoninic acid
CML	Nε-(carboxymethyl)lysine
Col I	type I collagen
CCK-8	Cell Counting Kit-8
ECM	extracellular matrix
ELISA	enzyme-linked immunosorbent assay
ELN	elastin
FC	fold change
FDR	false discovery rate
GO	glyoxal
HFF-1	human foreskin fibroblasts
LC-MS/MS	liquid chromatography-tandem mass spectrometry
MSI	Metabolomics Standards Initiative
NC	normal control
QC	quality control
OPLS-DA	orthogonal partial least squares discriminant analysis
VIP	variable importance in the projection
UHPLC	ultra-high-performance liquid chromatography coupled
